# Construction of fast xylose-fermenting yeast based on industrial ethanol-producing diploid *Saccharomyces cerevisiae* by rational design and adaptive evolution

**DOI:** 10.1186/1472-6750-13-110

**Published:** 2013-12-19

**Authors:** Liuyang Diao, Yingmiao Liu, Fenghui Qian, Junjie Yang, Yu Jiang, Sheng Yang

**Affiliations:** 1 CAS Key Laboratory of Synthetic Biology, Institute of Plant Physiology and Ecology, Shanghai Institutes for Biological Sciences, Chinese Academy of Sciences, 300 Fenglin Road, Shanghai 200032, China; 2 Shanghai Research and Development Center of Industrial Biotechnology, 528 Ruiqing Road, Shanghai 201201, China

**Keywords:** *Saccharomyces cerevisiae*, Xylose isomerase, Adaptive evolution, Xylose fermentation

## Abstract

**Background:**

It remains a challenge for recombinant *S. cerevisiae* to convert xylose in lignocellulosic biomass hydrolysates to ethanol. Although industrial diploid strains are more robust compared to laboratory haploid strains, however, industrial diploid *S. cerevisiae* strains have been less pursued in previous studies. This work aims to construct fast xylose-fermenting yeast using an industrial ethanol-producing diploid *S. cerevisiae* strain as a host.

**Results:**

Fast xylose-fermenting yeast was constructed by genome integration of xylose-utilizing genes and adaptive evolution, including 1) *Piromyces XYLA* was introduced to enable the host strain to convert xylose to xylulose; 2) endogenous genes (*XKS1, RKI1, RPE1, TKL1*, and *TAL1*) were overexpressed to accelerate conversion of xylulose to ethanol; 3) *Candida intermedia GXF1*, which encodes a xylose transporter, was introduced at the *GRE3* locus to improve xylose uptake; 4) aerobic evolution in rich xylose media was carried out to increase growth and xylose consumption rates. The best evolved strain CIBTS0735 consumed 80 g/l glucose and 40 g/l xylose in rich media within 24 hours at an initial OD_600_ of 1.0 (0.63 g DCW/l) and produced 53 g/l ethanol.

**Conclusions:**

Based on the above fermentation performance, we conclude that CIBTS0735 shows great potential for ethanol production from lignocellulosic biomass.

## Background

As a transportation fuel, ethanol has the potential to displace a substantial portion of gasoline. In recent years, ethanol production from lignocellulosic biomass has been attracting great attention. To produce ethanol, raw materials such as corn stover should be first pretreated and then hydrolyzed to liberate simple sugars, i.e. glucose and xylose. Subsequently, these sugars are converted to ethanol by microbial fermentation [1]. *Saccharomyces cerevisiae* is believed to be the most promising biocatalyst for this conversion due to its wide use in the starch- and sucrose-based ethanol industry [2]. However, *S. cerevisiae* cannot ferment xylose into ethanol. Since xylose is the second most abundant sugar present in the biomass hydrolysate after glucose, therefore fast xylose fermentation is required to produce ethanol from lignocellulosic biomass economically [3].

Great progress has been achieved to make *S. cerevisiae* able to ferment xylose in the last decade [4-8]. *S. cerevisiae* can take up xylose by nonspecific transporters [9]. After entering into cells, xylose can be converted to xylulose by either the oxidoreductive pathway or the isomerization pathway [2]. Since both pathways are absent in *S. cerevisiae*, therefore heterologous enzymes have to be introduced. Compared to the oxidoreductive pathway, the isomerization pathway receives more attention since it does not have a cofactor imbalance issue [8]. After phosphorylation by endogenous xylulokinase, xylulose enters the pentose phosphate pathway and then glycolysis to produce ethanol [10]. In previous studies, several steps have been identified as bottlenecks that limit the xylose consumption rate, including slow xylose uptake [11], slow conversion of xylose to xylulose [8], and limited flux of the pentose phosphate pathway [12]. Overexpression of xylose-utilizing proteins, to some extent, can remove these bottlenecks [5,11]. Moreover, *GRE3* is thought to cause xylitol accumulation and its deletion is beneficial to minimize xylitol formation [13]. As well as genetic manipulations, adaptive evolution is necessary to increase the xylose consumption rate. For example, several studies showed that combinatorial use of genetic manipulations (i.e. introduction of *XYLA* from *Piromyces* and overexpression of endogenous *XKS1*, *RPE1*, *RKI1*, *TAL1*, and *TKL1*) and adaptive evolution in xylose media can generate efficient xylose-utilizing strains [4,8,14]. However, there are two drawbacks for these studies: 1) laboratory haploid strains were chosen as hosts, which are generally considered not as robust as industrial diploid strains when fermenting lignocellulosic biomass hydrolysates [1] and 2) plasmid-based protein expression was employed, which is regarded as not stable as integration-based protein expression [15,16]. Although genome integration of xylose isomerase was pursued by Tanino et al [17], however, a laboratory haploid strain was selected to construct xylose-fermenting yeast. Until recently, a xylose-fermenting *S. cerevisiae* strain was reported based on an industrial diploid strain and genome integration of xylose isomerase and other genes [18].

So far, fermenting xylose in lignocellulosic biomass hydrolysates remains a challenge. Although industrial diploid strains are more robust compared to laboratory haploid strains, however, industrial diploid *S. cerevisiae* strains have been less pursued in previous studies. This study aims to construct fast xylose-fermenting yeast using an industrial ethanol-producing diploid *S. cerevisiae* strain as a host. For this purpose, *S. cerevisiae* CCTCC M94055 was chosen as the host. This strain is widely used to produce starch-based fuel and drinking ethanol and possesses phenotypes desired for industrial use, such as high tolerance to high temperatures, low pH value, and high ethanol and inhibitor concentrations [19,20]. To avoid unstable plasmid-based protein expression, we integrated all genes into chromosomes by homologous recombination. Specifically, in addition to introduction of *XYLA* and overexpression of *XKS1, RPE1, RKI1, TAL1*, *TKL1*, we also introduced a xylose transporter-encoding gene *GXF1* from *Candida intermedia* at the *GRE3* locus. After simple aerobic evolution in rich xylose media, the best evolved strain CIBTS0735 consumed 80 g/l glucose and 40 g/l xylose in 24 hours at an initial OD_600_ of 1.0 (0.63 g DCW/l) and produced 53 g/l ethanol.

## Results

### Rational construction of xylose-fermenting *S. cerevisiae*

**Figure 1:**
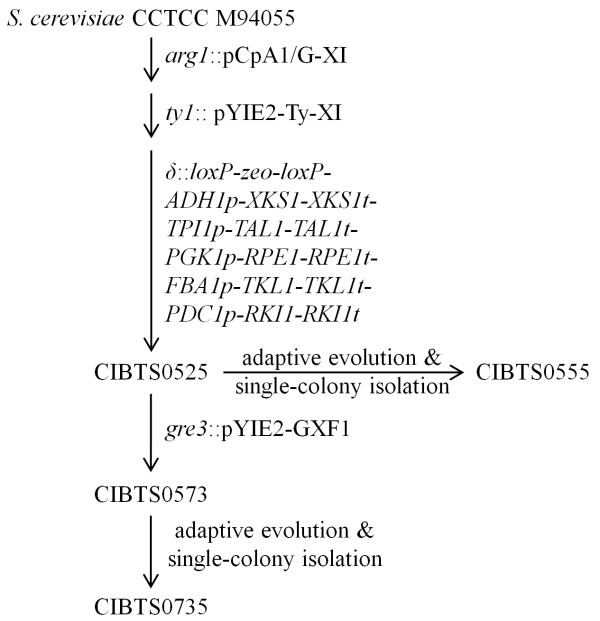
Schematic diagram of strain construction.

Figure 1 shows the whole process for strain construction. To convert xylose to xylulose in cells, two copies of a eukaryotic xylose isomerase-encoding gene *Piromyces XLYA* were integrated at the *ARG1* and *Ty1* loci sequentially. A strong promoter TPI1p was used to drive *XLYA* expression. Then, an additional copy of genes encoding xylulokinase (*XKS1*) and four non-oxidative enzymes of the pentose phosphate pathway (PPP, *RKI1, RPE1, TKL1*, and *TAL1*) were inserted at the *δ* locus, resulting in CIBTS0525. These genes were all equipped with strong promoters to achieve high expression and to accelerate conversion of xylulose to ethanol. Subsequently, a xylose transporter-encoding gene *GXF1* was integrated at the *GRE3* locus to increase the xylose uptake rate and the resultant strain was CIBTS0573. This integration inactivated one of the two copies of *GRE3*.

### Adaptive evolution to obtain fast xylose-fermenting *S. cerevisiae*

**Figure 2:**
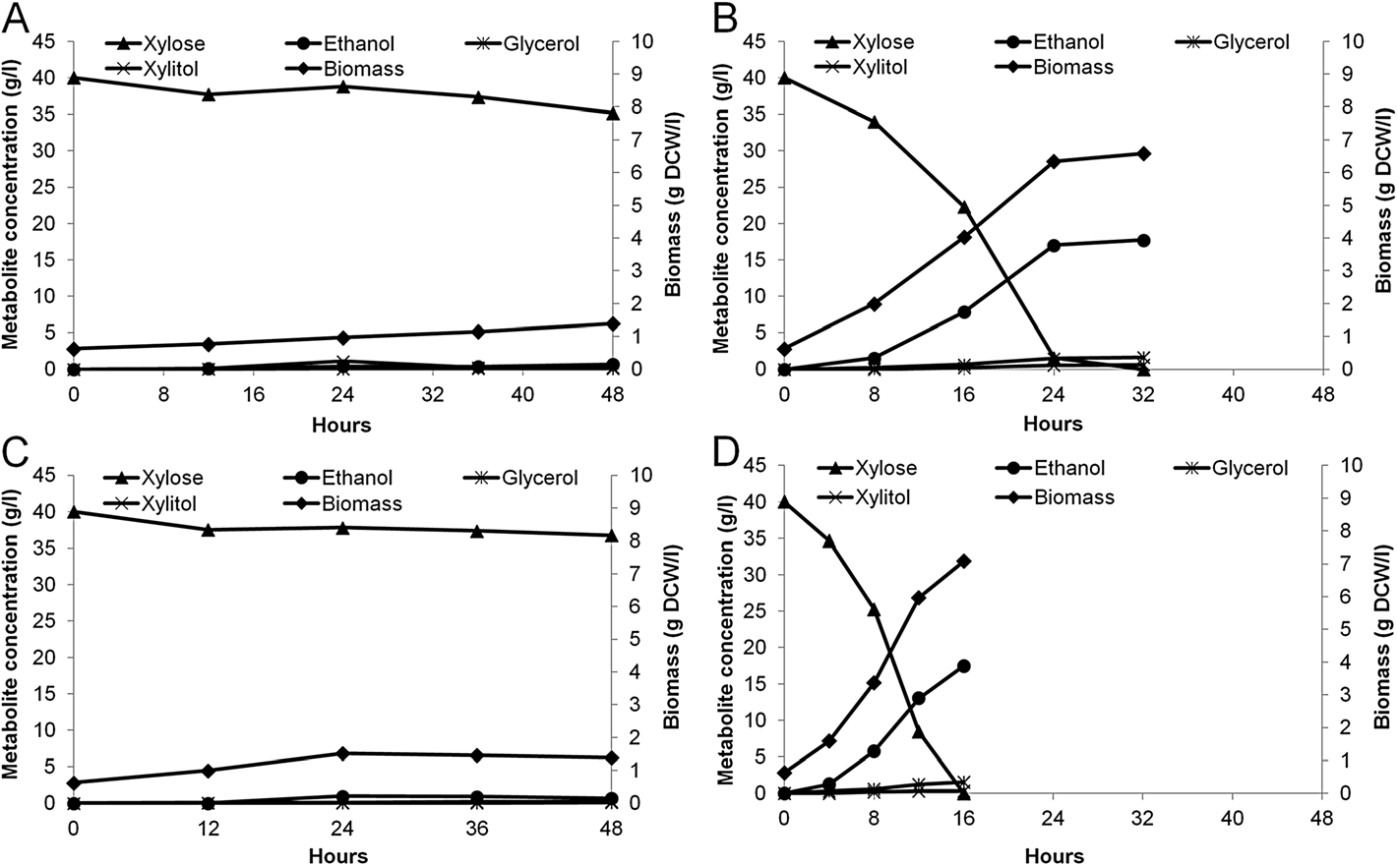
**Anaerobic fermentation of strains on xylose. (A)**, CIBTS0525; **(B)**, CIBTS0555; **(C)**, CIBTS0573; **(D)**, CIBTS0735. Strains were cultured in YP medium supplemented with 40 g/l xylose in shake flasks and the initial OD_600_ was set at 1.0 (0.63 g DCW/l). These fermentations were performed only once.

Although CIBTS0525 and CIBTS0573 contained a xylose-fermenting pathway, however both strains grew and consumed xylose slowly even under aerobic conditions (Figure 2 and Table 1). To select for spontaneous mutants with improved growth and xylose consumption rates, both strains were subjected to serial transfer in YP medium supplemented with xylose under aerobic conditions. After ca. 10 transfers, the growth and xylose consumption rates of both strains started to increase. Then additional several transfers were carried out for both strains until no improvement was observed. From the final transfers, both cultures were diluted and plated on YPX agar plates. Multiple colonies of each strain were inoculated into YP medium supplemented with 40 g/l xylose to examine their xylose consumption rates. The two best strains CIBTS0555 and CIBTS0735 were obtained from CIBTS0525 and CIBTS0573 respectively. As previously reported [8,14], these data show that adaptive evolution is required to obtain fast xylose-fermenting *S. cerevisiae* as well as metabolic engineering.

**Table 1:** **Fermentation performance of xylose-fermenting *S. cerevisiae *** **strains**

**Strain**	**CCTCC M94055**	**CIBTS0525**	**CIBTS0555**	**CIBTS0573**	**CIBTS0735**	**CIBTS0735**
Medium^a^	YPX40	YPX40	YPX40	YPX40	YPX40	YPD80X40
Specific growth rate^b^, h^-1^	N/A	0.016	0.096	0.019	0.187	0.181
Ethanol yield^c^, g/g	N/A	N/A	0.443	N/A	0.412	0.454
Glycerol yield^c^, g/g	N/A	N/A	0.039	N/A	0.039	0.041
Xylitol yield^c^, g/g	N/A	N/A	N/A	N/A	N/A	N/A
Sugar consumption rate^c^, g/g DCW/h	N/A	N/A	0.505	N/A	0.957	1.300
Ethanol production rate^c^, g/g DCW/h	N/A	N/A	0.224	N/A	0.394	0.590

^a^YPX40, YP medium supplemented with 40 g/l xylose; YPD80X40, YP medium supplemented with 80 g/l glucose and 40 g/l xylose. ^b^Specific growth rates were calculated from the exponential growth phase. ^c^Xylose consumption and ethanol production rates were calculated as described previously [21]. These two parameters as well as ethanol and xylitol yields were calculated from the growth phase (Figures 2 and 3, from the beginning of fermentation to below 10 g/l residual xylose).

Next, the two evolved strains CIBTS0555 and CIBTS0735 were characterized with respect to their growth and xylose fermentation rates in YP medium supplemented with 40 g/l xylose, using their unevolved parents as controls (Figure 2 and Table 1). For both the unevolved strains CIBTS0525 and CIBTS0573, cells grew very slowly with growth rates below 0.02 h^-1^ and also consumed xylose very slowly (Figure 2A, Figure 2C and Table 1). After adaptive evolution, in strikingly contrast, CIBTS0555 showed a 6-fold increase of the growth rate and its xylose consumption rate was increased to 0.505 g/g DCW/h (Figure 2B and Table 1). CIBTS0735 even displayed a 10-fold increase of the growth rate and its xylose consumption rate reached 0.957 g/g DCW/h (Figure 2D and Table 1). For both strains CIBTS0555 and CIBTS0735, ethanol yields exceeded 0.41 g/g xylose, while glycerol yields were kept low (below 0.04 g/g, Table 1) and no xylitol accumulation was detected. Taken together, these data clearly show that adaptive evolution significantly improves xylose fermentation and ethanol production.

### *GXF1* expression might contribute to increase growth and xylose consumption rates

A previous study showed that xylose uptake is a bottleneck for xylose fermentation and therefore, expression of *GXF1* encoding a xylose transporter can accelerate xylose utilization [11]. In this study *GXF1* also was introduced into CIBTS0525 to further increase the xylose consumption rate, resulting in CIBTS0573. Like CIBTS0525, CIBTS0573 also grew very slowly with a growth rate of 0.019 h^-1^ in YP medium supplemented with 40 g/l xylose. After adaptive evolution, compared to CIBTS0555 (evolved from CIBTS0525), CIBTS0735 (evolved from CIBTS0573) showed a 95% increase of the growth rate and a 90% increase of the xylose consumption rate (Figure 2D versus Figure 2B and Table 1). Accordingly, the ethanol production rate of CIBTS0735 was increased to 0.394 g/g DCW/h, which is 76% higher than that of CIBTS0555 (Figure 2D vs Figure 2B and Table 1). Besides, prolonged adaptive evolution of CIBTS0555 did not generate a strain displaying better growth and fermentation performance. These results imply that *GXF1* expression might contribute to increase growth and xylose consumption rates. Moreover, since CIBTS0555 and CIBTS0735 were evolved independently, we cannot exclude the possibility that different mutations might have occurred in the two evolved strains.

### Mixed sugars can be cofermented efficiently into ethanol

**Figure 3:**
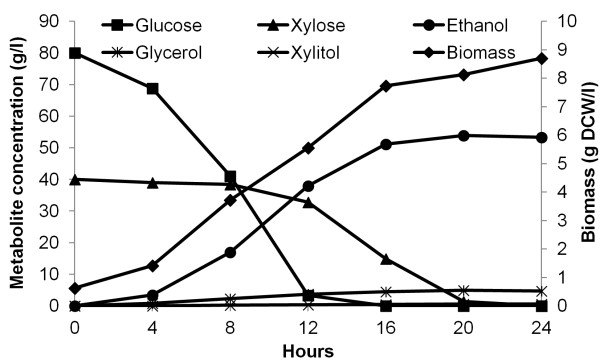
**Anaerobic fermentation of CIBTS0735 on mixed sugars.** CIBTS0735 was cultured in YP medium supplemented with 80 g/l glucose and 40 g/l xylose in a shake flask and the initial OD_600_ was set at 1.0 (0.63 g DCW/l). This fermentation was performed only once.

To test whether mixed sugars can be cofermented efficiently, the best evolved strain CIBTS0735 was characterized in YP medium supplemented with 80 g/l glucose and 40 g/l xylose. As shown in Figure 3, both sugars were consumed in 24 h with an ethanol yield of 0.45 g/g sugar. The total sugar consumption rate was 1.3 g/g DCW/h, which is 36% higher than that of CIBTS0735 cultivated in YP medium supplemented with 40 g/l xylose. However, although both sugars were utilized in 24 h, xylose was not significantly consumed until glucose was depleted. As for byproducts, glycerol yields were also kept low (0.041 g/g, Table 1) and no xylitol accumulation was detected. Taken together, although glucose and xylose were consumed sequentially, these data clearly show that mixed sugars can be cofermented efficiently into ethanol.

### CIBTS0735 exhibits a higher activity of xylose isomerase

**Figure 4:**
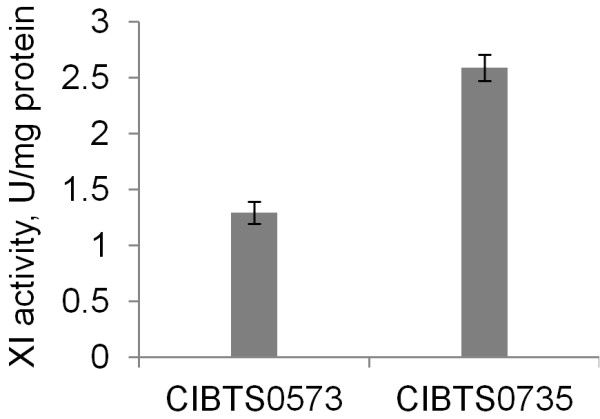
**Xylose isomerase (XI) activities of CIBTS0573 and CIBTS0735.** The averages of three replicates for each strain and standard deviations are presented.

A recent study showed that an increased activity of xylose isomerase was observed during adaptive evolution, which in part contributed to efficient xylose assimilation [8]. In this study, we also examined whether the activity of xylose isomerase had increased during the adaptive evolution of CIBTS0735. To do this, xylose isomerase activities of strains CIBTS0573 and CIBTS0735 were compared. As shown in Figure 4, after adaptive evolution, a 100% increase of the xylose isomerase activity was observed for CIBTS0735. This result suggests that the elevated activity of xylose isomerase might have contributed to the efficient xylose fermentation of CIBTS0735.

## Discussion

Fast xylose fermentation is a key technology to produce ethanol from lignocellulosic biomass economically. Although great progress has been achieved in the last decade, however xylose fermentation remains a challenge today. Based on an industrial ethanol-producing diploid strain *S. cerevisiae* CCTCC M94055, we constructed fast xylose-fermenting *S. cerevisiae* by genome integration of xylose-utilizing genes in this study.

Strong promoter-driven expression of *XYLA*, *XKS1*, and four genes of the non-oxidative PPP did not immediately result in obvious growth on xylose. For example, aerobic growth rates of CIBTS0525 and CIBTS0573 were 0.016 h^-1^ and 0.019 h^-1^, respectively. Although these two strains grew at rates comparable to similarly constructed strains H131-A3 (growth rate, 0.031 h^-1^) [8] and BSPC095 (growth rate, 0.009 h^-1^) [14], their growth rates were substantially lower than that of RWB217 (growth rate, 0.22 h^-1^) [5]. This might be caused by 1) only two copies of *XYLA* integrated into the genome and 2) a different host strain used. By adapting CIBTS0525 on xylose, we obtained CIBTS0555 that showed a growth rate of 0.096 h^-1^ in rich xylose media. In parallel, we also engineered another industrial ethanol-producing diploid *S. cerevisiae* strain CICC 1300 using the same strategy as CCTCC M94055 (Additional file 1: Figure S1). The resultant strain CIBTS0552 displayed a comparable growth rate (0.097 h^-1^) to that of CIBTS0555, suggesting that the engineering strategy is applicable to not only laboratory strains [5,8,14], but industrial strains.

Previous studies showed that *GXF1* expression is beneficial to xylose fermentation in *S. cerevisiae*[6,11,22,23] and these studies were all carried out using recombinant yeast strains with a limited flux of xylose metabolism. In this study, we show that *GXF1* expression might contribute to increase growth and xylose consumption rates. This notion could be further supported by additional evidence. In parallel to *GXF1*, we also introduced into CIBTS0525 another xylose transporter-encoding gene *SUT1* from *Pichia stipitis* (Additional file 1: Figure S2). After adaptive evolution, the resultant strain (CIBTS0734) exhibited a xylose consumption rate of 0.800 g/g DCW/h in rich xylose media, which is 58% higher than that of CIBTS0555 (0.505 g/g DCW/h). However, without introducing heterologous xylose transporters, no further improvement was observed for CIBTS0555 through prolonged adaptation on xylose. Besides, these results also suggest that xylose uptake is a bottleneck for CIBTS0555 that displays a relatively high flux of xylose consumption.

It has been reported that anaerobic xylose-limited continuous cultivation in a chemostat was required to reach maximal growth rates, as well as (an)aerobic sequential batch cultivation [6]. However, in this study only simple aerobic batch cultivation was employed to generate fast-growing CIBTS0735 with a growth rate of 0.187 h^-1^ in rich xylose media. The reason for dispensability of chemostat cultivation is probably attributed to *GXF1* expression, since the purpose of xylose-limited chemostat cultivation is to improve xylose uptake [4,8] which is the same as the purpose of *GXF1* expression.

Table 2 compares several best-performing xylose-fermenting strains. From this overall comparison, we know that CIBTS0735 displays comparable or even superior phenotypes. However, it should be noted that the fermentation for these strains was carried out under different conditions. Moreover, CIBTS0735 was also examined in the undetoxified corn stover hydrolysate provided by National Renewable Energy Laboratory and Novozymes A/S. Although under harsh conditions (e.g. 8.4 g/l acetic acid and 2.6 g/l HMF), CIBTS0735 still converted 82.3 g/l glucose and 46.7 g/l xylose to 51.8 g/l ethanol (Additional file 1: Figure S3). This result suggests that CIBTS0735 is a potential biocatalyst for ethanol production from lignocellulosic biomass. However, compared to the xylose fermentation in the rich media, the xylose utilization in the corn stover hydrolysate was much slower, which was probably caused by the presence of high concentrations of inhibitors. Rational design and adaptation against inhibitors may be pursued to increase xylose fermentation in the undetoxified corn stover hydrolysate [24-28].

**Table 2:** **Performance of metabolically engineered *S*. *cerevisiae *** **strains in anaerobic batch cultivation**

**Strain**	**Description**^**a**^	**Condition**	**Initial sugar, g/l**	**Final ethanol**^**b,c**^**, g/l**	**Y**_**E**_^**b,c**^**, g/g**	**Y**_**G**_^**b,c**^**, g/g**	**R**_**S**_^**b,c**^**, g/l/h**	**R**_**E**_^**b,c**^**, g/l/h**	**Reference**
RWB218	*piXYLA*; *XKS1*; *TAL1*; *TKL1*; *RKI1*; *RPE1*; Δ*GRE3*; adaptive evolution	Defined medium; inoculum size at 1.1 g DCW/l; fermentation time at 24 h	100 (glu.) 25 (xyl.)	47.5	0.38	0.08	5.21	1.98	[4]
H131-A3-AL^CS^	*piXYLA*; *psXYL3*; *psTAL1*; *TKL1*; *RPE1*; *RKI1*; *ARG4*; *LEU2*; adaptive evolution	Defined medium; inoculum size at 0.05 g DCW/l; fermentation time at ca. 30 h	40 (xyl.)	16.4	0.41	N/A	1.33	0.55	[8]
GS1.11-26	*cpxylA*; *XKS1*; *TAL1*; *TKL1*; *RPE1*; *RKI1*; *HXT7*; *TKL2*; *TAL2*; *psARAT*, *blaraB*; *ecaraA*; *ecaraD*; adaptive evolution	Rich medium; inoculum size at1.3 g DCW/l; fermentation time at 13 h	36 (glu.) 37 (xyl.)	33.6	0.46	N/A	5.62	2.58	[18]
CIBTS0735	*piXYLA*; *XKS1*; *TAL1*; *TKL1*; *RKI1*; *RPE1*; *ciGXF1*; adaptive evolution	Rich medium; inoculum size at 0.63 g DCW/l; fermentation time at 16 h	40 (xyl.)	17.47	0.44	0.04	2.50	1.09	This study
		Rich medium; inoculum size at 0.63 g DCW/l; fermentation time at 24 h	80 (glu.) 40 (xyl.)	53.34	0.44	0.04	5.00	2.22	This study

^a^*piXYLA*, *Piromyces XYLA*; *psXYL3*, *Pichia stipitis XYL3*; *psTAL1*, *P. stipitis TAL1*; *cpxylA*, *Clostridium phytofermentans xylA*; *psARAT*, *P. stipitis ARAT*; *blaraB*, *Bacillus subtilis araB*; *ecaraA*, *Escherichia coli araA*; *ecaraD*, *E. coli araD*; *ciGXF1*, *Candida intermedia GXF1*. ^b^Y_E_, ethanol yield; Y_G_, glycerol yield; R_S_, sugar consumption rate; R_E_, ethanol production rate. ^c^Values for all strains were recalculated based on the whole fermentation process.

Evolutionary engineering is a powerful tool to generate strains with desired production traits [29]. Several studies employed this tool to generate improved xylose-fermenting yeast [4,8,14,30]. However, this technique provides no information on genetic changes. For CIBTS0735, the increased activity of xylose isomerase is likely to contribute to its fast growth and efficient xylose fermentation. Previous studies also observed an increase of the xylose isomerase activity after adaptive evolution [8,14]. Except for the elevated xylose isomerase activity, other mutations might also have occurred in CIBTS0735 to account for improved phenotypes, including increased xylose transport [4], balanced xylose metabolic flux [8], and even genome-scale changes [8,14]. However, accurate genetic changes occurred in CIBTS0735 remain to be elucidated by genome resequencing and transcriptome analysis and this work will be pursued in our following research.

## Conclusions

Here we report the construction of fast xylose-fermenting yeast CIBTS0735 based on xylose isomerase and industrial diploid *S. cerevisiae*. This strain can convert 80 g/l glucose and 40 g/l xylose to 53 g/l ethanol in 24 hours at initial OD_600_ of 1.0 (0.63 g DCW/l) and therefore shows great potential for fuel ethanol production from lignocellulosic biomass.

## Methods

### Strains and plasmids

Strains and plasmids used in this study are listed in Table 3. *Escherichia coli* DH5α was used as a cloning host. *S. cerevisiae* CCTCC M94055 was used as the host strain to construct xylose-fermenting yeast.

**Table 3:** Strains and plasmids used in this study

**Strain/plasmid**	**Description**	**Reference**^**a**^
CCTCC M94055	An industrial ethanol-producing *S. cerevisiae* strain; *MAT*a*/α*	CCTCC
CIBTS0525	*S. cerevisiae* CCTCC M94055 derivative; *arg1*::pCpA1/G-XI; *ty1*::pYIE2-Ty-XI; *δ*::*loxP-zeo-loxP-ADH1p-XKS1-XKS1t-TPI1p-TAL1-TAL1t-PGK1p-RPE1-RPE1t-FBA1p-TKL1-TKL1t-PDC1p-RKI1-RKI1t*	This study
CIBTS0555	A single-colony isolate of CIBTS0525 after adaptive evolution	This study
CIBTS0573	CIBTS0525 derivative; *gre3*::pYIE2-GXF1	This study
CIBTS0735	A single-colony isolate of CIBTS0573 after adaptive evolution	This study
pCpA1/G-XI	Carrying *TPI1p-XYLA-CYC1t*, used to integrate a copy of *Piromyces XYLA* at the *ARG1* locus	This study
pYIE2-Ty-XI	Carrying *TPI1p-XYLA-CYC1t*, used to integrate a copy of *Piromyces XYLA* at the *Ty1* locus	This study
pSH47-hph	pSH47 encoding a hygromycin-resistant protein	This study
pYIE2-GXF1	Carrying *TPI1p-GXF1-TPI1t*, used to integrate *Candida intermidia GXF1* at the *GRE3* locus	This study

^a^CCTCC, China Center for Type Culture Collection.

### Media and culture conditions

*E. coli* strains were grown in LB medium at 37°C, 250 rpm and when necessary, 100 μg/ml ampicillin was supplemented for plasmid propagation. *S. cerevisiae* strains were grown in YP medium (20 g/l tryptone and 10 g/l yeast extract) supplemented with glucose (YPD), xylose (YPX), or glucose and xylose (YPDX) at 30°C, 250 rpm and when necessary, antibiotics were added as follows: G418, 200 μg/ml; zeocin, 200 μg/ml; hygromycin, 200 μg/ml.

### Plasmid construction

Plasmids were constructed by conventional cloning methods. pCpA1/G-XI was constructed to integrate a copy of *Piromyces XYLA* encoding xylose isomerase at the *ARG1* locus and pYIE2-Ty-XI was constructed to integrate the second *XYLA* copy at the *Ty1* locus. For both plasmids, TPI1p (promoter of *TPI1*) and CYC1t (terminator of *CYC1*) were used to drive *XYLA* expression. Genetic maps of these two plasmids are shown in Additional file 1: Figure S4. pSH47-hph was constructed to rescue *loxP*-flanked selection markers by cloning a hygromycin-resistant gene into pSH47 [31]. pYIE2-GXF1 was constructed to integrate *Candida intermedia GXF1* that encodes a xylose transporter at the *GRE3* locus. For *GXF1*, TPI1p and TPI1t (terminator of *TPI1*) were used to drive gene expression. The genetic map of pYIE2-GXF1 is shown in Additional file 1: Figure S5.

### Strain construction

All *S. cerevisiae* strains were constructed from *S. cerevisiae* CCTCC M94055. The process of strain construction was depicted in Figure 1. The first *XYLA* copy was integrated at the *ARG1* locus by transforming Kpn2I-linearized pCpA1/G-XI using G418 as the selection marker and similarly, the second *XYLA* copy was integrated at the *Ty1* locus by transforming SalI-linearized pYIE2-Ty-XI using zeocin as the selection marker. Then, zeocin rescue was performed as previously described using pSH47-hph [31]. Subsequently, a DNA fragment (Additional file 1: Figure S6) containing *loxP-zeo-loxP*, *ADH1p-XKS1-XKS1t*, *TPI1p-TAL1-TAL1t*, *PGK1p-RPE1-RPE1t*, *FBA1p-TKL1-TKL1t*, and *PDC1p-RKI1-RKI1t* was directly assembled and inserted at the *δ* locus using DNA Assembler [32] and zeocin as the selection marker. After zeocin rescue, pYIE2-GXF1 was linearized by PstI and then transformed into CIBTS0525 to integrate *GXF1* at the *GRE3* locus, resulting in CIBTS0573.

### Adaptive evolution by serial transfer

For adaptive evolution, strains were grown aerobically in YP medium supplemented with 20–40 g/l xylose. When cultures entered into the stationary phase, new cultivations were made by transferring 10% (v/v) of the cultures into fresh media. This procedure was repeated ca. 15 cycles until the xylose consumption rate did not increase any more. Finally, cultures were diluted and plated on YPX agar plates for single-colony isolation.

### Anaerobic fermentation in rich media

Strains were first grown aerobically in 5 ml YP medium supplemented with 20 g/l glucose and 10 g/l xylose in 20 ml test tubes overnight at 30°C, 250 rpm. Then, cultures were used to inoculate 30 ml fresh media in 250 ml flasks. After aerobic growth, cells from the late stationary phase were harvested, washed twice with sterile water, and later used to inoculate 100 ml YP medium supplemented with 40 g/l xylose or 80 g/l glucose and 40 g/l xylose in 300 ml bottles capped with rubber stoppers (syringe needles were inserted into rubber stoppers to release CO_2_ during fermentation) at an initial OD_600_ of 1.0 (0.63 g DCW/l). During fermentation, samples were taken at intervals for analysis of OD_600_ and metabolites.

### Analytical methods

Cell densities (OD_600_) were determined using Beckman Coulter DU 730 Spectrophotometer. For determination of cell dry weight, different volumes (0.5 ml - 1.5 ml) of cell cultures were collected by centrifugation. Cells were washed once with sterile water, dried in a 105°C oven for 48 h, and then weighed. One OD_600_ unit corresponded to 0.63 g DCW/l. Ethanol was detected using Agilent 7890A GC with an Alltech EC-WAX column and a flame ionization detector. The column was eluted at 85°C with nitrogen. Glucose, xylose, glycerol, and xylitol were detected using Agilent 1200 HPLC with a Bio-Rad HPX-87H column and a refractive index detector. The column was eluted at 65°C with 5 mM of sulfuric acid at a flow rate of 0.6 ml/min.

### Enzyme assay

Strains were grown anaerobically to the early stationary phase in YP medium supplemented with 20 g/l glucose and 10 g/l xylose. Cells were harvested by centrifugation at room temperature and washed twice with sterile water. After suspended in Tris–HCl buffer (50 mM, pH7.5), cells were disrupted by sonication. Cell debris was removed by centrifugation and crude extracts were reserved for enzyme assays. The total protein concentration in cell extracts was determined using the Bradford assay with bovine serum albumin as the standard. The activity of xylose isomerase was measured in reaction mixtures containing 50 mM phosphate buffer (pH6.8), 1 mM MnCl_2_, 5 mM xylose, and cell extracts. After incubation at 37°C for 20 min, xylulose was quantified by the cysteine-carbazole-sulfuric acid method [33].

## Competing interests

The authors declare that they have no competing interests.

## Authors’ contributions

LD, JY, YJ and SY designed the study, interpreted the results and wrote the manuscript. YL and FQ performed the experiments. All authors read and approved the final manuscript.

## Supplementary Material

Additional file 1 **Supplemental material. Figure S1**, Anaerobic fermentation of CIBTS0552 on xylose. (A), Construction process of CIBTS0552. (B), Xylose fermentation. The strain was cultured at 30°C with shaking in YP medium supplemented with 40 g/l xylose in a 300 ml shake flask containing 100 ml medium. The shake flask was capped with a rubber stopper (a syringe needle was inserted into the rubber stopper to release CO_2_ during fermentation). The initial OD_600_ was set at 1.0 (0.63 g DCW/l). **Figure S2**, Anaerobic fermentation of CIBTS0734 on xylose. (A), Construction process of CIBTS0734. (B), Xylose fermentation. The strain was cultured at 30°C with shaking in YP medium supplemented with 40 g/l xylose in a 300 ml shake flask containing 100 ml medium. The shake flask was capped with a rubber stopper (a syringe needle was inserted into the rubber stopper to release CO_2_ during fermentation). The initial OD_600_ was set at 1.0 (0.63 g DCW/l). **Figure S3**, Anaerobic fermentation of CIBTS0735 in undetoxified corn stover hydrolysate. The strain was cultured at 30°C with shaking in a 100 ml shake flask containing 30 ml undetoxified corn stover hydrolysate. The hydrolysate was only supplemented with 1 g/l urea and its pH was adjusted to 6.0. The shake flask was capped with a rubber stopper (a syringe needle was inserted into the rubber stopper to release CO_2_ during fermentation) and the initial OD_600_ was set at 8.0 (5 g DCW/l). The hydrolysate contained 82.3 g/l glucose, 54.2 g/l xylose, 8.4 g/l acetic acid and 2.6 g/l HMF. **Figure S4**, Genetic maps for pCpA1/G-XI and pYIE2-Ty-XI. **Figure S5**, Genetic map for pYIE2-GXF1. **Figure S6**, DNA fragment used to overexpress xylulokinase and the four nonoxidative enzymes in the pentose phosphate pathway at the *δ* locus. Click here for file

## References

[B1] Hahn-Hägerdal B Karhumaa K Fonseca C Spencer-Martins I Gorwa-Grauslund MF Towards industrial pentose-fermenting yeast strains Appl Microbiol Biotechnol 2007 13 937 953 10.1007/s00253-006-0827-2 17294186

[B2] Matsushika A Inoue H Kodaki T Sawayama S Ethanol production from xylose in engineered *Saccharomyces cerevisiae* strains: current state and perspectives Appl Microbiol Biotechnol 2009 13 37 53 10.1007/s00253-009-2101-x 19572128

[B3] Kim SR Ha SJ Wei N Oh EJ Jin YS Simultaneous co-fermentation of mixed sugars: a promising strategy for producing cellulosic ethanol Trends Biotechnol 2012 13 274 282 10.1016/j.tibtech.2012.01.005 22356718

[B4] Kuyper M Toirkens MJ Diderich JA Winkler AA van Dijken JP Pronk JT Evolutionary engineering of mixed-sugar utilization by a xylose-fermenting *Saccharomyces cerevisiae* strain FEMS Yeast Res 2005 13 925 934 10.1016/j.femsyr.2005.04.004 15949975

[B5] Kuyper M Hartog MMP Toirkens MJ Almering MJH Winkler AA van Dijken JP Pronk JT Metabolic engineering of a xylose-isomerase-expressing *Saccharomyces cerevisiae* strain for rapid anaerobic xylose fermentation FEMS Yeast Res 2005 13 399 409 10.1016/j.femsyr.2004.09.010 15691745

[B6] Runquist D Hahn-Hagerdal B Rådström P Comparison of heterologous xylose transporters in recombinant *Saccharomyces cerevisiae* Biotechnol Biofuels 2010 13 5 10.1186/1754-6834-3-5 20236521 PMC2851583

[B7] Sedlak M Ho NWY Production of ethanol from cellulosic biomass hydrolysates using genetically engineered *Saccharomyces* yeast capable of cofermenting glucose and xylose Appl Biochem Biotechnol 2004 13 403 416 15054267 10.1385/abab:114:1-3:403

[B8] Zhou H Cheng JS Wang BL Fink GR Stephanopoulos G Xylose isomerase overexpression along with engineering of the pentose phosphate pathway and evolutionary engineering enable rapid xylose utilization and ethanol production by *Saccharomyces cerevisiae* Metab Eng 2012 13 611 622 10.1016/j.ymben.2012.07.011 22921355

[B9] Hamacher T Becker J Gárdonyi M Hahn-Hägerdal B Boles E Characterization of the xylose-transporting properties of yeast hexose transporters and their influence on xylose utilization Microbiology 2002 13 2783 2788 12213924 10.1099/00221287-148-9-2783

[B10] van Maris AJ Abbott DA Bellissimi E van den Brink J Kuyper M Luttik MA Wisselink HW Scheffers WA van Dijken JP Pronk JT Alcoholic fermentation of carbon sources in biomass hydrolysates by *Saccharomyces cerevisiae*: current status Antonie Van Leeuwenhoek 2006 13 391 418 10.1007/s10482-006-9085-7 17033882

[B11] Runquist D Fonseca C Rådström P Spencer-Martins I Hahn-Hägerdal B Expression of the Gxf1 transporter from *Candida intermedia* improves fermentation performance in recombinant xylose-utilizing *Saccharomyces cerevisiae* Appl Microbiol Biotechnol 2009 13 123 130 10.1007/s00253-008-1773-y 19002682

[B12] Johansson B Hahn-Hägerdal B The non-oxidative pentose phosphate pathway controls the fermentation rate of xylulose but not of xylose in *Saccharomyces cerevisiae* TMB3001 FEMS Yeast Res 2002 13 277 282 12702276 10.1111/j.1567-1364.2002.tb00095.x

[B13] Träff KL Cordero RRO van Zyl WH Hahn-Hägerdal B Deletion of the *GRE3* aldose reductase gene and its influence on xylose metabolism in recombinant strains of *Saccharomyces cerevisiae* expressing the *xylA* and *XKS1* genes Appl Environ Microbiol 2001 13 5668 5674 10.1128/AEM.67.12.5668-5674.2001 11722921 PMC93358

[B14] Shen Y Chen X Peng B Chen L Hou J Bao X An efficient xylose-fermenting recombinant *Saccharomyces cerevisiae* strain obtained through adaptive evolution and its global transcription profile Appl Microbiol Biotechnol 2012 13 1079 1091 10.1007/s00253-012-4418-0 23053078

[B15] Meinander NQ Hahn-Hägerdal B Fed-batch xylitol production with two recombinant *Saccharomyces cerevisiae* strains expressing *XYL1* at different levels, using glucose as a cosubstrate: a comparison of production parameters and strain stability Biotechnol Bioeng 1997 13 391 399 10.1002/(SICI)1097-0290(19970520)54:4<391::AID-BIT12>3.0.CO;2-J 18634106

[B16] Zhang Z Moo-Young M Chisti Y Plasmid stability in recombinant *Saccharomyces cerevisiae* Biotechnol Adv 1996 13 401 435 10.1016/S0734-9750(96)00033-X 14540156

[B17] Tanino T Hotta A Ito T Ishii J Yamada R Hasunuma T Ogino C Ohmura N Ohshima T Kondo A Construction of a xylose-metabolizing yeast by genome integration of xylose isomerase gene and investigation of the effect of xylitol on fermentation Appl Microbiol Biotechnol 2010 13 1215 1221 10.1007/s00253-010-2870-2 20853104

[B18] Demeke MM Dietz H Li Y Foulquié-Moreno MR Mutturi S Deprez S Den Abt T Bonini BM Liden G Dumortier F Development of a D-xylose fermenting and inhibitor tolerant industrial *Saccharomyces cerevisiae* strain with high performance in lignocellulose hydrolysates using metabolic and evolutionary engineering Biotechnol Biofuels 2013 13 89 10.1186/1754-6834-6-89 23800147 PMC3698012

[B19] Huang H Guo X Li D Liu M Wu J Ren H Identification of crucial yeast inhibitors in bio-ethanol and improvement of fermentation at high pH and high total solids Bioresour Technol 2011 13 7486 7493 10.1016/j.biortech.2011.05.008 21624827

[B20] Kang D Zhao L Comparison of the fermentation performance of several strains of achohol yeast Liquor-making Sci Technol 2006 13 40 43

[B21] Peng B Shen Y Li X Chen X Hou J Bao X Improvement of xylose fermentation in respiratory-deficient xylose-fermenting *Saccharomyces cerevisiae* Metab Eng 2012 13 9 18 10.1016/j.ymben.2011.12.001 22178745

[B22] Parachin NS Bergdahl B van Niel EW Gorwa-Grauslund MF Kinetic modelling reveals current limitations in the production of ethanol from xylose by recombinant *Saccharomyces cerevisiae* Metab Eng 2011 13 508 517 10.1016/j.ymben.2011.05.005 21642010

[B23] Tanino T Ito T Ogino C Ohmura N Ohshima T Kondo A Sugar consumption and ethanol fermentation by transporter-overexpressed xylose-metabolizing *Saccharomyces cerevisiae* harboring a xyloseisomerase pathway J Biosci Bioeng 2012 13 209 211 10.1016/j.jbiosc.2012.03.004 22591844

[B24] Fujitomi K Sanda T Hasunuma T Kondo A Deletion of the *PHO13* gene in *Saccharomyces cerevisiae* improves ethanol production from lignocellulosic hydrolysate in the presence of acetic and formic acids, and furfural Bioresour Technol 2012 13 161 166 22357292 10.1016/j.biortech.2012.01.161

[B25] Hasunuma T Sung KM Sanda T Yoshimura K Matsuda F Kondo A Efficient fermentation of xylose to ethanol at high formic acid concentrations by metabolically engineered *Saccharomyces cerevisiae* Appl Microbiol Biotechnol 2011 13 997 1004 10.1007/s00253-011-3085-x 21246355

[B26] Koppram R Albers E Olsson L Evolutionary engineering strategies to enhance tolerance of xylose utilizing recombinant yeast to inhibitors derived from spruce biomass Biotechnol Biofuels 2012 13 32 10.1186/1754-6834-5-32 22578262 PMC3408370

[B27] Liu ZL Molecular mechanisms of yeast tolerance and in situ detoxification of lignocellulose hydrolysates Appl Microbiol Biotechnol 2011 13 809 825 10.1007/s00253-011-3167-9 21380517

[B28] Wright J Bellissimi E de Hulster E Wagner A Pronk JT van Maris AJ Batch and continuous culture-based selection strategies for acetic acid tolerance in xylose-fermenting *Saccharomyces cerevisiae* FEMS Yeast Res 2011 13 299 306 10.1111/j.1567-1364.2011.00719.x 21251209

[B29] Sauer U Evolutionary engineering of industrially important microbial phenotypes Adv Biochem Eng Biotechnol 2001 13 129 169 11816810 10.1007/3-540-45300-8_7

[B30] Kuyper M Winkler AA van Dijken JP Pronk JT Minimal metabolic engineering of *Saccharomyces cerevisiae* for efficient anaerobic xylose fermentation: a proof of principle FEMS Yeast Res 2004 13 655 664 10.1016/j.femsyr.2004.01.003 15040955

[B31] Güldener U Heck S Fiedler T Beinhauer J Hegemann JH A new efficient gene disruption cassette for repeated use in budding yeast Nucleic Acids Res 1996 13 2519 2524 10.1093/nar/24.13.2519 8692690 PMC145975

[B32] Shao Z Zhao H DNA assembler, an in vivo genetic method for rapid construction of biochemical pathways Nucleic Acids Res 2009 13 e16 10.1093/nar/gkn724 19074487 PMC2632897

[B33] Dische Z Borenfreund E A new spectrophotometric method for the detection and determination of keto sugars and trioses J Biol Chem 1951 13 583 587 14907652

