# Supplementary material for: Construction of fast xylose-fermenting yeast based on industrial ethanol-producing diploid Saccharomyces cerevisiae by rational design and adaptive evolution
Source: BMC Biotechnol. 2013 Dec 19;13:110. doi: 10.1186/1472-6750-13-110 (PMC3878346; doi:10.1186/1472-6750-13-110)
Supplement: Additional file 1 — Supplemental material. Figure S1, Anaerobic fermentation of CIBTS0552 on xylose. (A), Construction process of CIBTS0552. (B), Xylose fermentation. The strain was cultured at 30°C with shaking in YP medium supplemented with 40 g/l xylose in a 300 ml shake flask containing 100 ml medium. The shake flask was capped with a rubber stopper (a syringe needle was inserted into the rubber stopper to release CO2 during fermentation). The initial OD600 was set at 1.0 (0.63 g DCW/l). Figure S2, Anaerobic fermentation of CIBTS0734 on xylose. (A), Construction process of CIBTS0734. (B), Xylose fermentation. The strain was cultured at 30°C with shaking in YP medium supplemented with 40 g/l xylose in a 300 ml shake flask containing 100 ml medium. The shake flask was capped with a rubber stopper (a syringe needle was inserted into the rubber stopper to release CO2 during fermentation). The initial OD600 was set at 1.0 (0.63 g DCW/l). Figure S3, Anaerobic fermentation of CIBTS0735 in undetoxified corn stover hydrolysate. The strain was cultured at 30°C with shaking in a 100 ml shake flask containing 30 ml undetoxified corn stover hydrolysate. The hydrolysate was only supplemented with 1 g/l urea and its pH was adjusted to 6.0. The shake flask was capped with a rubber stopper (a syringe needle was inserted into the rubber stopper to release CO2 during fermentation) and the initial OD600 was set at 8.0 (5 g DCW/l). The hydrolysate contained 82.3 g/l glucose, 54.2 g/l xylose, 8.4 g/l acetic acid and 2.6 g/l HMF. Figure S4, Genetic maps for pCpA1/G-XI and pYIE2-Ty-XI. Figure S5, Genetic map for pYIE2-GXF1. Figure S6, DNA fragment used to overexpress xylulokinase and the four nonoxidative enzymes in the pentose phosphate pathway at the δ locus. [file 1472-6750-13-110-S1.docx]

**Supplemental material**

Figure S1, Anaerobic fermentation of CIBTS0552 on xylose. (A), Construction process of CIBTS0552. (B), Xylose fermentation. The strain was cultured at 30 ^o^C with shaking in YP medium supplemented with 40 g/l xylose in a 300 ml shake flask containing 100ml medium. The shake flask was capped with a rubber stopper (a syringe needle was inserted into the rubber stopper to release CO_2_ during fermentation). The initial OD_600_ was set at 1.0 (0.63 g DCW/l).

Figure S2, Anaerobic fermentation of CIBTS0734 on xylose. (A), Construction process of CIBTS0734. (B), Xylose fermentation. The strain was cultured at 30 ^o^C with shaking in YP medium supplemented with 40 g/l xylose in a 300 ml shake flask containing 100ml medium. The shake flask was capped with a rubber stopper (a syringe needle was inserted into the rubber stopper to release CO_2_ during fermentation). The initial OD_600_ was set at 1.0 (0.63 g DCW/l).

Figure S3, Anaerobic fermentation of CIBTS0735 in undetoxified corn stover hydrolysate. The strain was cultured at 30 ^o^C with shaking in a 100 ml shake flask containing 30ml undetoxified corn stover hydrolysate. The hydrolysate was only supplemented with 1 g/l urea and its pH was adjusted to 6.0. The shake flask was capped with a rubber stopper (a syringe needle was inserted into the rubber stopper to release CO_2_ during fermentation) and the initial OD_600_ was set at 8.0 (5 g DCW/l). The hydrolysate contained 82.3 g/l glucose, 54.2 g/l xylose, 8.4 g/l acetic acid and 2.6 g/l HMF.


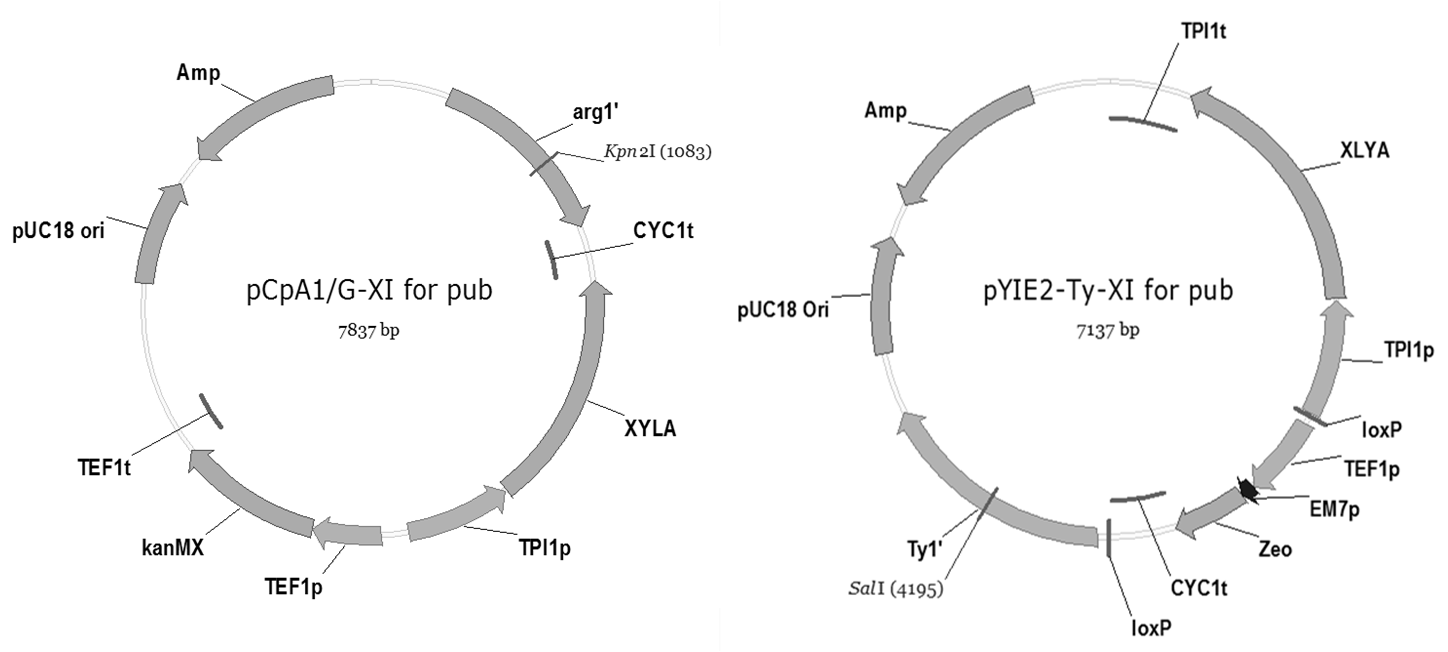


Figure S4, Genetic maps for pCpA1/G-XI and pYIE2-Ty-XI


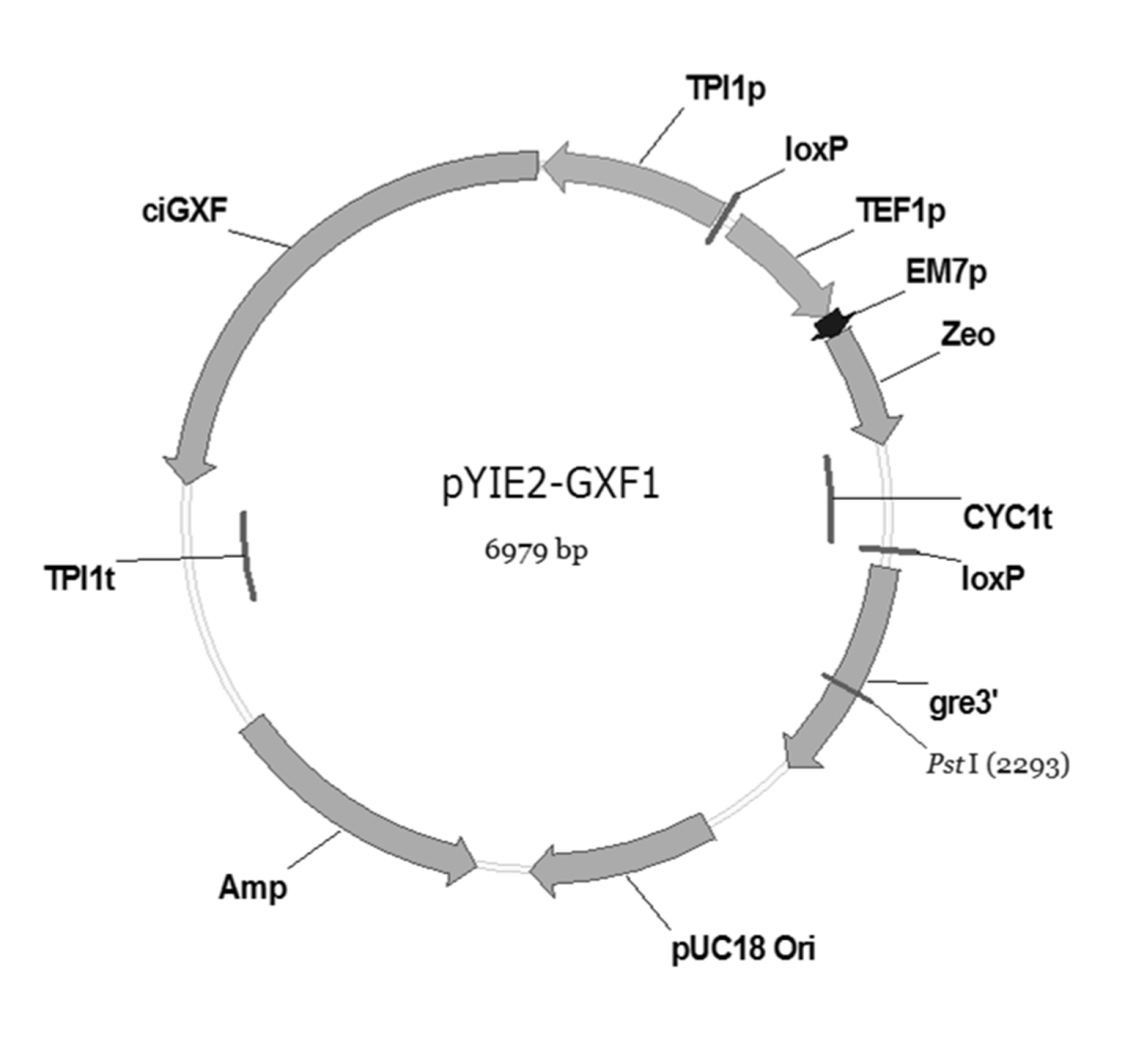


Figure S5, Genetic map for pYIE2-GXF1


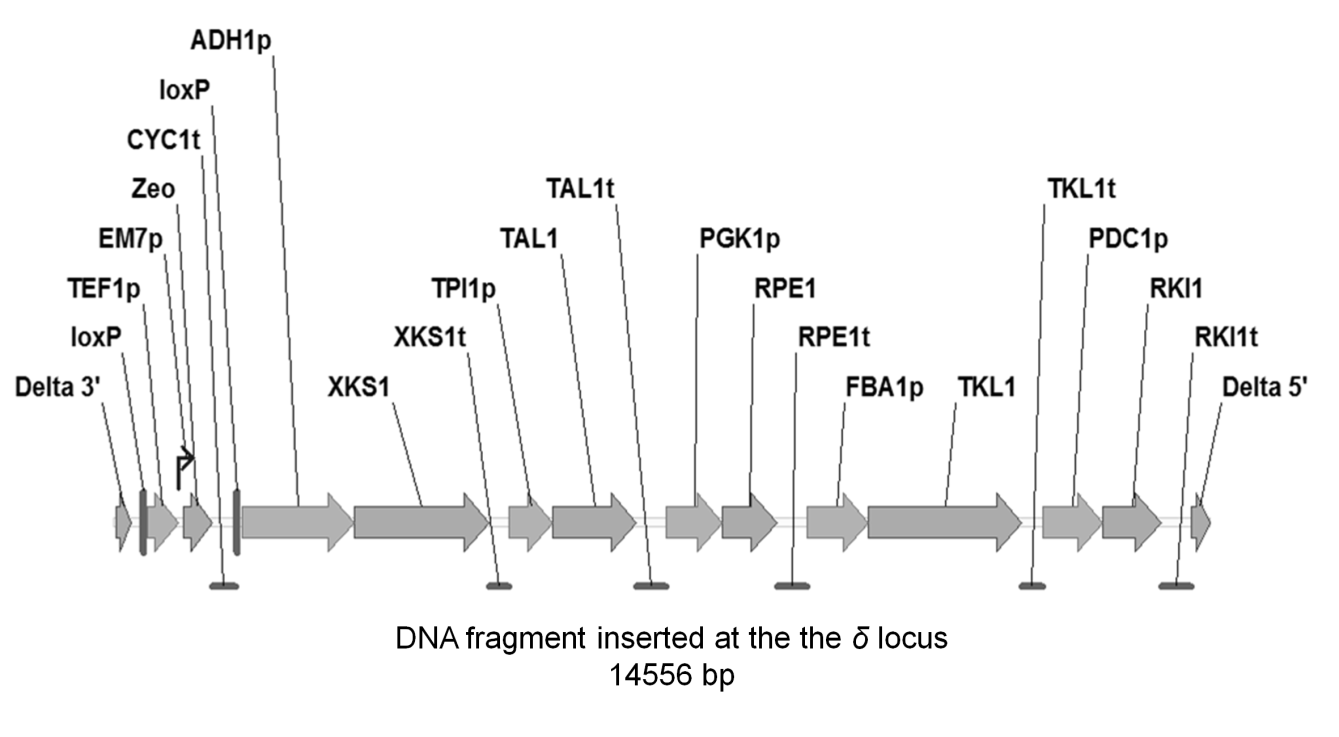
Figure S6, DNA fragment used to overexpress xylulokinase and the four nonoxidative enzymes in the pentose phosphate pathway at the *δ* locus.
